# Do not attempt resuscitation orders at the emergency department of a teaching hospital

**DOI:** 10.1590/S1679-45082017AO3999

**Published:** 2017

**Authors:** Cássia Regina Vancini-Campanharo, Rodrigo Luiz Vancini, Marcelo Calil Machado, Maria Carolina Barbosa Teixeira Lopes, Meiry Fernanda Pinto Okuno, Ruth Ester Assayag Batista, Aécio Flávio Teixeira de Góis

**Affiliations:** 1 Universidade Federal de São Paulo, São Paulo, SP, Brazil.; 2 Universidade Federal do Espírito Santo, Espírito Santo, ES, Brazil.; 3 Hospital Nipo-Brasileiro, São Paulo, SP, Brazil.

**Keywords:** Heart arrest, Cardiopulmonary resuscitation/ethics, Resuscitation orders, Decision making/ethics, Emergency service, hospital, Parada cardíaca, Reanimação cardiopulmonar/ética, Ordens quanto à conduta (Ética Médica), Tomada de decisões/ética, Serviço hospitalar de emergência

## Abstract

**Objective:**

To identify factors associated with not attempting resuscitation.

**Methods:**

A cross-sectional study conducted at the emergency department of a teaching hospital. The sample consisted of 285 patients; in that, 216 were submitted to cardiopulmonary resuscitation and 69 were not. The data were collected by means of the in-hospital Utstein Style. To compare resuscitation attempts with variables of interest we used the χ^2^ test, likelihood ratio, Fisher exact test, and analysis of variance (p<0.05).

**Results:**

No cardiopulmonary resuscitation was considered unjustifiable in 56.5% of cases; in that, 37.7% did not want resuscitation and 5.8% were found dead. Of all patients, 22.4% had suffered a previous cardiac arrest, 49.1% were independent for Activities of Daily Living, 89.8% had positive past medical/surgical history; 63.8% were conscious, 69.8% were breathing and 74.4% had a pulse upon admission. Most events (76.4%) happened at the hospital, the presumed cause was respiratory failure in 28.7% and, in 48.4%, electric activity without pulse was the initial rhythm. The most frequent cause of death was infection. The factors that influenced non-resuscitation were advanced age, history of neoplasm and the initial arrest rhythm was asystole.

**Conclusion:**

Advanced age, past history of neoplasia and asystole as initial rhythm were factors that significantly influenced the non-performance of resuscitation. Greater clarity when making the decision to resuscitate patients can positively affect the quality of life of survivors.

## RESUMO

**Objetivo:**

Identificar fatores associados à não realização de ressuscitação.

**Métodos:**

Estudo transversal realizado no serviço de emergência de um hospital universitário. A amostra foi composta por 285 pacientes, dos quais 216 foram submetidos à ressuscitação cardiopulmonar, e 69 não tiveram esta conduta. Os dados foram coletados por meio do *in-hospital Utstein Style*. Para comparar as tentativas de ressuscitação e as variáveis de interesse, utilizaram-se o teste do χ^2^, a razão de verossimilhança, o teste exato de Fisher e a análise de variância (p<0,05).

**Resultados:**

A não ressuscitação foi considerada injustificável em 56,5% dos casos, sendo que 37,7% não queriam ressuscitação e 5,8% foram encontrados mortos. Do total de pacientes, 22,4% tiveram parada cardíaca prévia, 49,1% eram independentes para Atividades de Vida Diária, e 89,8% tinham alguma história pregressa; 63,8% estavam conscientes, 69,8% estavam respirando e 74,4% tinham pulso palpável à admissão. A maioria dos eventos (76,4%) ocorreu no hospital, e a causa presumida de parada foi insuficiência respiratória em 28,7% e, em 48,4%, o ritmo inicial foi atividade elétrica sem pulso. A causa mais frequente de morte foi infecção. Os fatores que influenciaram na não realização de ressuscitação foram idade avançada, história de neoplasia e assistolia como ritmo inicial de parada.

**Conclusão:**

Idade avançada, história de neoplasia e assistolia como ritmo inicial foram os fatores que influenciaram significativamente na não realização de ressuscitação. Maior clareza na decisão de reanimar pode afetar positivamente a qualidade de vida dos pacientes.

## INTRODUCTION

It is estimated that 200,000 individuals are attended at the emergency departments in Brazil every year due to cardiac arrest, and half of them occur in hospital settings. Mortality data are scarce though.^(^ ^1^ ^)^

Cardiac arrest is a severe and often fatal problem, and its reversion demands immediate cardiopulmonary resuscitation (CPR).^(^ ^2^ ^)^ When CPR is applied to patients beyond the possibilities of cure, death can be postponed, and the state of persistent coma can be determined, with disastrous consequences for the patient, family and community.^(^ ^3^ ^)^

In Brazil, the do not attempt resuscitation orders have no legal support, obliging healthcare professionals to apply CPR in all situations, except if death is obvious. This imposes the need for standardized conducts, taking into account moral and ethical aspects of each situation, aiming for the individual's wellbeing.^(^ ^2^ ^)^

Emergency treatment is needed when an individual is confronted with the real possibility of death, severe body injury or deterioration of health status, resulting from a sudden, unexpected situation and not from a chronic and incurable disease.^(^ ^4^ ^)^ Nevertheless, the decision not to resuscitate can be difficult for the health team, due to a lack of research in this area, lack of formal training to recognize these situations, and differences in attitudes and personal values.^(^ ^5^ ^)^

In some cases, CPR can result in unexpected consequences that are worse than death. For patients, it can cause physical discomfort and an unacceptable quality of life; for family members, it can entail false hope and extremely high costs; for professionals involved, feelings of frustration and sadness and, for society, exaggerated resource consumption.^(^ ^6^ ^)^

Anticipated guidelines for end-of-life care and the documentation of the patient's preferences in these circumstances can minimize this situation, reduce the number of unnecessary reanimations, keep the patient's dignity and reduce suffering of family members and healthcare team.^(^ ^6^ ^)^

## OBJECTIVE

To identify possible factors associated with no attempt to perform cardiopulmonary resuscitation.

## METHODS

It is cross-sectional study, undertaken at the emergency department of a teaching hospital that provided high-complexity care. This study was approved by the Institutional Review Board at the *Universidade Federal de São Paulo* (protocol number 0030/2011).

The study population consisted of patients seen at the emergency department and diagnosed as cardiac arrest, confirmed based on unconsciousness, absence of breathing and central pulse. A convenience sample was obtained between January 1st, 2011 and January 31st, 2012. The inclusion criteria were cardiac arrest patients at or outside the hospital, seen at the emergency department during the study period. The exclusion criteria were cardiac arrest patients seen at other units of the hospital.

**Figure 1:**
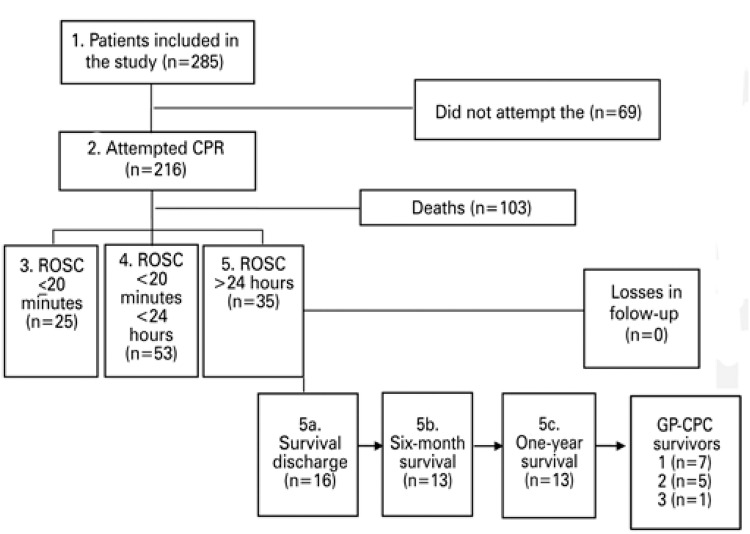
Flowchart of patients in the study CPR: cardiopulmonary resuscitation; ROSC: return of spontaneous circulation; GP-CPC: Glasgow-Pittsburgh Cerebral Performance Category

In this study, CPR was defined as the application of Basic Life-Support maneuvers (ventilation, external thoracic compression and defibrillation) and/or Advanced Life-Support maneuvers (tracheal intubation and medication). Figure 1 presents the patient's flowchart.

Trained nurses collected the data, using the *In-hospital Utstein Style*,^(^ ^7^ ^)^ a standard report to collect significant data in cardiac arrest.

The following sociodemographic and clinical variables studied were sex, age, skin color, pre-cardiac arrest neurological condition, past medical/surgical history, level of consciousness, breathing and pulse upon admission to emergency department. The variables related to the cardiac arrest were place of occurrence, presence of witnesses, presumed immediate cause, initial rhythm of cardiac arrest, CPR attempt and cause of death.

To analyze the data, the software Statistical Package for the Social Sciences (SPSS) version 19 was used. For patients with more than one cardiac arrest, the data for the first event were collected. Frequency and percentage were used for categorical variables. Number of valid observations (n), mean and standard deviation (SD) were presented for continuous numerical variables. To compare the CPR attempts with categorical variables (sex, skin color, past history, conscious upon admission, breathing upon admission, pulse present upon admission, pre-cardiac arrest Cerebral Performance Category (CPC), place of event, witnesses, immediate cause, initial rhythm and cause of death), the χ^2^ test was used. When the values of 20% or more of the boxes were less than five, the odds ratio or Fisher's exact test were used. To compare the CPR attempts with continuous variable (age), Analysis of Variance (ANOVA) was used, after verifying homogeneity of the variances. The level of significance considered was 5% (p value<0.05).

## RESULTS

The sample consisted of 285 patients, of which 216 were submitted to CPR. Among the patients who were not submitted to CPR (n=69), in 56.5%, the CPR was considered unjustifiable, 37.7% did not want resuscitation and, in 5.8% of the cases, the patients were found dead.

The demographic and clinical data of the study population are presented o table 1. The past history of the study population are presented on table 2. The cardiac arrest characteristics of the study population are presented on table 3.

**Table 1:** Demographic and clinical variables of the study population

Variables		Resuscitation attempt		Total 100%
		Yes n (%)	No n (%)	
Sex				
	Male	127 (79.9)	32(20.1)	159
	Female	89 (70.6)	37 (29.4)	126
	Total	216 (75.8)	69 (24.2)	285
Skin color				
	White	154 (75.1)	51 (24.9)	205
	Black	23 (76.7)	7 (23.3)	30
	Yellow	8 (66.7)	4 (33.3)	12
	Brown	31(81.6)	7 (18.4)	38
	Total	216 (75.8)	69 (24.2)	285
Conscient upon admission				
	Yes	134 (73.6)	48 (26.4)	182
	No	82 (79.6)	21 (20.4)	103
	Total	216 (75.8)	69 (24.2)	285
Breathing upon admission				
	Yes	141 (70.9)	58(29.1)	199
	No	75(87.2)	11 (12.8)	86
	Total	216 (75.8)	69 (24.2)	285
Pulse present upon admission				
	Yes	150 (70.8)	62 (29.2)	212
	No	66(90.4)	7 (9.6)	73
	Total	216 (75.8)	69 (24.2)	285
GP-CPC pre-cardiac arrest				
	1	69 (93.2)	5 (6.8)	74
	2	104 (74.3)	36 (25.7)	140
	3	37 (60.7)	24 (39.3)	61
	4 and 5	2(40.0)	3 (60.0)	5
	Total	212 (75.7)	68 (24.3)	280
Age				
	Mean±SD	65.1 ± 17.4	70.0 ± 16.1	66.3 ± 17.2
	Total	216	69	285

GP-CPC: Glasgow-Pittsburgh Cerebral Performance Category; SD: standard deviation.

**Table 2:** Personal antecedents of the study population

Variables	Resuscitation Attempt		Total (n=285)
	Yes (n=216)	No (n=69)	
Psychiatric disease	6 (100.0)		6
Neurological disease	24 (70.6)	10 (29.4)	34
Dementia	12 (63.2)	7 (36.8)	19
Pulmonary disease	5 (100.0)		5
Arrhythmia	15 (88.2)	2(11.8)	17
Myocardiopathy	26 (78.8)	7(21.2)	33
Liver disease	9 (69.2)	4 (30.8)	13
Kidney disease	15 (83.3)	3 (16.7)	18
Neoplasms	44 (56.4)	34 (43.6)	78
Arterial hypertension	95 (79.2)	25 (20.8)	120
Dyslipidemia	17 (94.4)	1 (5.6)	18
*Diabetes mellitus*	49 (87.5)	7 (12.5)	56
Smoking	21 (84.0)	4 (16.0)	25
Alcohol	5 (62.5)	3 (37.5)	8
Others	14 (82.4)	3(17.6)	17

**Table 3:** Characteristics of the cardiac arrest of the study population

Variables		Resuscitation attempt		Total 100%
		Yes n (%)	No n (%)	
Place of event				
	Out of hospital	59 (88.1)	8(11.9)	67
	At the hospital	217(71.1)	88 (28.9)	305
	Total	276 (74.2)	96 (25.8)	372
Witnessed				
	Sim	249 (73.2)	91 (26.8)	340
	Não	27 (84.4)	5 (15.6)	32
	Total	276 (74.2)	96 (25.8)	372
Immediate cause				
	Lethal arrhythmia	13 (92.9)	1 (7.1)	14
	Hypotension	45 (65.2)	24 (34.8)	69
	Respiratory failure	72 (75.0)	24 (25.0)	96
	Metabolic alteration	69 (73.4)	25 (26.6)	94
	Ischemia or stroke	53 (96.4)	2 (3.6)	55
	Other	7 (29.2)	17 (70.8)	24
	Total	259 (73.6)	93 (26.4)	352
Initial rhythm				
	Ventricular fibrillation	16 (94.1)	1 (5.9)	17
	Ventricular tachycardia	3 (50.0)	3 (50.0)	6
	Asystole	49(45.4)	59 (54.6)	108
	Pulseless electrical activity	166 (86.9)	25 (13.1)	191
	Total	234 (72.7)	88 (27.3)	322

Table 4 presents the factors that were significantly associated with the CPR attempts of the study population.

**Table 4:** Factors associated with attempts at cardiopulmonary resuscitation in the study population

Variables		Resuscitation attempt		Total	p value
		Yes n (%)	No n (%)		
Breathing upon admission					0.0031*
	Yes	141 (70.9)	58(29.1)	199	
	No	75 (87.2)	11 (12.8)	86	
	Total	216 (75.8)	69 (24.2)	285	
Pulse present upon admission					0.0007*
	Yes	150 (70.8)	62 (29.2)	212	
	No	66 (90.4)	7 (9.6)	73	
	Total	216	69	285	
GP-CPC pre-cardiac arrest					<0.0001*
	1	69 (93.2)	5 (6.8)	74	
	2	104 (74.3)	36 (25.7)	140	
	3	37 (60.7)	24 (39.3)	61	
	4 and 5	2 (40.0)	3 (60.0)	5	
	Total	212 (75.7)	68 (24.3)	280	
Past history of neoplasm					<0,0001*
	No	172 (83.1)	35 (16.9)	207	
	Yes	44 (56.4)	34 (43.6)	78	
	Total	216 (75.8)	69 (24.2)	285	
*Diabetes mellitus*					0.0225*
	No	167 (72.9)	62 (27.1)	229	
	Yes	49 (87.5)	7 (12.5)	56	
	Total	216 (75.8)	69 (24.2)	285	
Place of event					0.0042*
	Out of hospital	59 (88.1)	8(11.9)	67	
	At hospital	217 (71.1)	88 (28.9)	305	
	Total	276 (74.2)	96 (25.8)	372	
Immediate cause					<0.0001*
	Lethal arrhythmia	13 (92.9)	1 (7.1)	14	
	Hypotension	45 (65.2)	24 (34.8)	69	
	Respiratory failure	72 (75.0)	24 (25.0)	96	
	Metabolic alteration	69 (73.4)	25 (26.6)	94	
	Ischemia or stroke	53 (96.4)	2 (3.6)	55	
	Other	7 (29.2)	17 (70.8)	24	
	Total	259 (73.6)	93 (26.4)	352	
Initial rhythm					<0.0001*
	Ventricular fibrillation	16 (94.1)	1 (5.9)	17	
	Ventricular tachycardia	3 (50.0)	3 (50.0)	6	
	Asystole	49(45.4)	59 (54.6)	108	
	Pulseless electrical activity	166 (86.9)	25 (13.1)	191	
	Total	234 (72.7)	88 (27.3)	322	
Age					0.0378†
	Mean±SD	65.1±17.4	70.0±16.1	66.3 (17.2)	
	Total	216	69	285	

* χ^2^ test;

† analysis of variance.

CPC: Glasgow-Pittsburgh Cerebral Performance Category; SD: standard deviation.

The factors that were significantly associated with high rates of CPR attempts were as follows: breathing and pulse present upon admission; low scores in the pre-cardiac arrest CPC; absence of neoplasms, past history of *diabetes mellitus;* cardiac arrest outside the hospital; arrhythmia and ischemia/stroke as a presumed cause of cardiac arrest, and ventricular fibrillation and pulseless electrical activity as the initial cardiac arrest rhythm.

Patients of more advanced age, with other factor as a presumed cause of cardiac arrest and with asystole as initial cardiac arrest rhythm were less submitted to CPR attempts.

## DISCUSSION

Although some years have gone since the publication of studies on do not attempt resuscitation orders, inconsistency remains in the interpretation of these orders, which can cause confusion regarding the clinical situations they apply, and difficulties in terms of defining the best end-of-life care.^(^ ^5^ ^,^ ^7^ ^)^

These difficulties are even greater in cases of cardiac arrest because the events can occur suddenly, without knowing the patients’ history. In addition, do not attempt resuscitation orders refer to the accomplishment or not of CPR efforts, without considering pre-existing diseases, the objectives of care and the patient's desires.^(^ ^7^ ^)^

In this study, the factors that significantly influenced the accomplishment or not of CPR were advanced age, past history, neurological status before cardiac arrest, place of cardiac arrest, breathing and pulse upon admission, immediate cause and initial rhythm of cardiac arrest, and cause of death.

Also, patients of more advanced age were less frequently submitted to CPR attempts. A multicenter study undertaken in the United States to verify the influence of post-cardiac arrest care in patients aged over 75 years demonstrated that these elderly were more prone to do not attempt resuscitation orders (65.9% *versus* 48.2%, p<0.001) and the removal of Advanced Life-Support measures (61.2% *versus* 47.5%, p=0.005). However, after 6-month follow-up, the survivors preserved a favorable neurological status (CPC 1 and 2), similar to younger patients.^(^ ^8^ ^)^

The advanced age seems to influence the nonresuscitation orders,^(^ ^9^ ^)^ which may be associated with the presence of more comorbidities and a bad quality of life after CPR in this population.^(^ ^8^ ^)^ Nevertheless, the results appoint that age as an isolated factors for non-resuscitation decisions may not be reliable, as the treatment targets should be considered, as well as the fact that the higher mortality may be related to the suspension of advanced care, and even to the do not attempt resuscitation orders attributed to these individuals.^(^ ^8^ ^,^ ^10^ ^)^

As regards to past history, patients with a history of *diabetes mellitus* and no history of cancer showed a higher percentage of CPR attempts. A prospective study of 1,446 patients at a university hospital in Geneva, aiming to determine the prevalence of do not attempt resuscitation orders and related factors, among medical professionals, evidenced that the presence of cancer diagnosis was associated with more do not attempt resuscitation order prescribed by these professionals. Most decisions, however, were made instinctively, with no clear treatment objectives and goals,^(^ ^9^ ^)^ which may suggest that the meaning, objectives and application of the non-resuscitation orders should be systematically discussed at the universities that offer healthcare programs, presented as protocols and documented at the organizations, entailing safer decision-making and care for these patients.^(^ ^9^ ^)^

In this study, patients with lower pre-cardiac arrest CPC levels showed a higher percentage of CPR attempts. Some studies indicated poor quality of life before and after CPR as an independent factor associated with do not attempt resuscitation orders. In addition, patients with cognitive deficits may not be capable of participating in the decisions on their health status. The use of scales to assess the patient neurological status and quality of life can minimize this issue.^(^ ^9^ ^)^

In the study population, patients who were victims of cardiac arrest out of hospital and who presented breathing or pulse upon admission showed a higher percentage of CPR attempts. This finding can be attributed to the sudden nature of the cardiac arrest and to the professionals’ lack of knowledge on these patients’ medical history. In addition, in cases of out- of-hospital cardiac arrest, care may be first provided by laymen, who are not skilled and authorized to decide about do not attempt resuscitation orders.^(^ ^11^ ^,^ ^12^ ^)^

In the present study, patients with ventricular fibrillation or pulseless electrical activity as the initial cardiac arrest rhythm showed a higher percentage of CPR attempts, whereas those with asystole showed a higher percentage of no CPR attempt.

The do not attempt resuscitation orders may have been less common in patients with ventricular fibrillation, as this initial cardiac arrest rhythm was associated with a greater probability of return to spontaneous circulation and better long-term neurological outcomes - provided that CPR and early defibrillation are provided.^(^ ^8^ ^,^ ^13^ ^,^ ^14^ ^)^

In the patients for whom arrhythmia, ischemia or stroke were the presumed immediate causes, the percentage of CPR attempts was higher. This finding may be related to the fact that cardiovascular disease is the main cause of death in the adult population around the world, and it is associated with sudden events, such as stroke, ventricular arrhythmias and cardiac arrest, in which the patient's medical history is unknown, with no consensus and security in application of do not attempt resuscitation orders.^(^ ^15^ ^)^

Death is one of the most controversial issues in modern society, for cultural, economic and social reasons. Healthcare professionals also demonstrate difficulties when dealing with this situation, often because they are not prepared for their own death.^(^ ^10^ ^)^

In this context, the do not attempt resuscitation orders should be reconsidered in accordance with the ethical principles of beneficence and non-maleficence, engaging the patients and all people who effectively take care of them, with clear objectives and goals for patients receiving end-of-life care, and providing for a dignified death, with as much and timely comfort as possible.^(^ ^10^ ^)^

In many cases, CPR has no physiological utility, that is, it may not offer benefits to cure the underlying disease. However, it is useful in legal terms, for taking into account the patient's perspective. More informed decision-making on whether to reanimate or not can positively affect the survivors’ quality of life.

The difference between do not attempt resuscitation orders and the objectives and goals of end-of-life care to patients with untreatable diseases is not clear yet.^(^ ^9^ ^,^ ^16^ ^)^ The professionals often make this decision based on each person's life experience and knowledge level, which can impede care delivery to certain patients.^(^ ^5^ ^)^

The results of this study can help elaborate specific guidelines for do not attempt resuscitation orders, clarifying information to the population about the legitimacy of these orders, and allowing patients to use this right to care and good practices.^(^ ^10^ ^)^

The main limitation of this study was the small number of patients, but its strengths were to be performed at a university reference and high-complexity hospital, and the prospective data collection, which involved consecutive patients.

## CONCLUSION

Of the patients who were not submitted to cardiopulmonary resuscitation, in the majority of the cases, this procedure was considered unjustifiable. The factors that significantly influenced the non-accomplishment of cardiopulmonary resuscitation were patient's advanced age, past history of cancer, and asystole as the initial cardiac arrest rhythm.
